# Scaphocapitate arthrodesis with lunate preservation for Kienböck’s disease: prospective outcomes study

**DOI:** 10.1007/s00402-024-05423-1

**Published:** 2024-07-15

**Authors:** Galal Hegazy, Amro A. Fouaad, Rashed Emam El-Sadek, Emad Zayed, Yasser Saqr, Ehab Alshal

**Affiliations:** 1 Orthopedic Department, Faculty of Medicine, AL-Azhar University, Nasr City, Cairo, 11884 Egypt; 2 Orthopedic Department, Faculty of Medicine, Portsaid University, Portfouad, Portsaid, 42526 Egypt; 3 Orthopedic Department, Faculty of Medicine, AL-Azhar University, Assiut City, Assiut, 71524 Egypt

**Keywords:** Kienböck’s disease, Scaphocapitate, Arthrodesis, Intercarpal arthrodesis, Fusion, Wrist

## Abstract

**Purpose:**

The study evaluated the efficacy of SC arthrodesis with lunate preservation for treating patients diagnosed with stage IIIB or IIIC Kienböck’s disease, who also exhibit neutral ulnar variance. The study further aimed to explore potential variations in outcomes between patients diagnosed with stage IIIB and IIIC Kienböck’s disease.

**Methods:**

Thirty-two patients diagnosed with stage IIIB (*n* = 19) and stage IIIC (*n* = 13) Kienböck’s disease underwent SC arthrodesis with distal radius bone grafting stabilised by Herbert compression screws. All participants underwent pre- and post-operative assessments including VAS score for pain, ROM, grip strength, MMWS, and the Quick DASH score. Additionally, RS angle, LHI ratio, and CHI ratio were assessed.

**Results:**

For all patients, the mean operative time was 73 min, follow-up was 45.6 months, time to union was 14 weeks, and time to full return to work was 24 weeks. The rate of union at the arthrodesis site was 91% (29 out of 32 patients) whilst the incidence of postoperative degenerative arthritis was 36% (8 out of 32 patients). Regarding changes in the means of outcomes from pre- to post-operatively, the VAS score decreased from 8.2 to 1.3 and grip strength improved from 36 to 79%. The RS angle was corrected from 59° to 50°. Significant improvements were noted in the mean MMWS from 45 to 75 and QuickDASH score from 78 to 21. However, no significant changes were observed in ROM, LHI, and CHI. There were no significant differences between patients with stage IIIB and stage IIIC in terms of these parameters, except for differences observed in the RS angle, LHI, and CHI preoperatively and in LHI and CHI postoperatively.

**Conclusion:**

Evidence level: II.

Our research demonstrates that SC arthrodesis is a valuable approach for reducing pain, improving grip strength, and enhancing overall function in individuals with advanced Kienböck’s disease. Importantly, our results indicate no notable differences in outcomes between patients diagnosed with stage IIIB or IIIC Kienböck’s disease.

## Introduction

Kienböck’s disease is characterised by a compromised blood supply to the lunate bone, leading to its collapse and potential fragmentation, resulting in pain, stiffness, and progressive arthritis in the wrist [1–3]. The modified Lichtman classification categorises the disease into four stages. Stage I displays normal X-rays, although MRI may reveal early changes. Stage II is recognised by increased bone density but without alteration in shape or fractures. Stage III, which is more severe, is further divided into IIIA (lunate collapse without carpal misalignment), IIIB (lunate collapse with decreased carpal height), and IIIC (coronal fracture of the lunate). Stage IV indicates advanced disease with significant arthritis [4].

The management of Kienböck’s disease varies from simple immobilisation to more complex surgical interventions including lunate off-loading and joint levelling, revascularisation procedures to enhance blood supply, and salvage techniques for advanced stages. Scaphocapitate (SC) arthrodesis, a salvage surgical technique involving limited inter-carpal fusion, provides a motion-preserving alternative [1]. This procedure aims to stabilise the wrist and redistribute stress away from the radiolunate (RL) joint, thereby alleviating strain on the lunate bone [2, 3]. Although the current literature predominantly relies on retrospective analyses to evaluate the effectiveness of SC arthrodesis, a scarcity of prospective studies has been noted [5–9].

This prospective study seeks to assess the clinical and radiographic outcomes of SC arthrodesis in patients with stage IIIB and IIIC Kienböck’s disease according to the modified Lichtman classification [4]. Additionally, the study will investigate potential variations in outcomes between patients with stage IIIB and IIIC Kienböck’s disease before and after surgery.

## Materials and methods

Our institutional review board approved the study prior to commencement, adhering to the ethical guidelines of the Helsinki Declaration of 1975, as revised in 2000 and 2008. From December 2017 to February 2024, the hand surgery clinic at our university hospitals enrolled 73 consecutive patients with Kienböck’s disease to evaluate their eligibility for participation in the study.

**Fig. 1:**
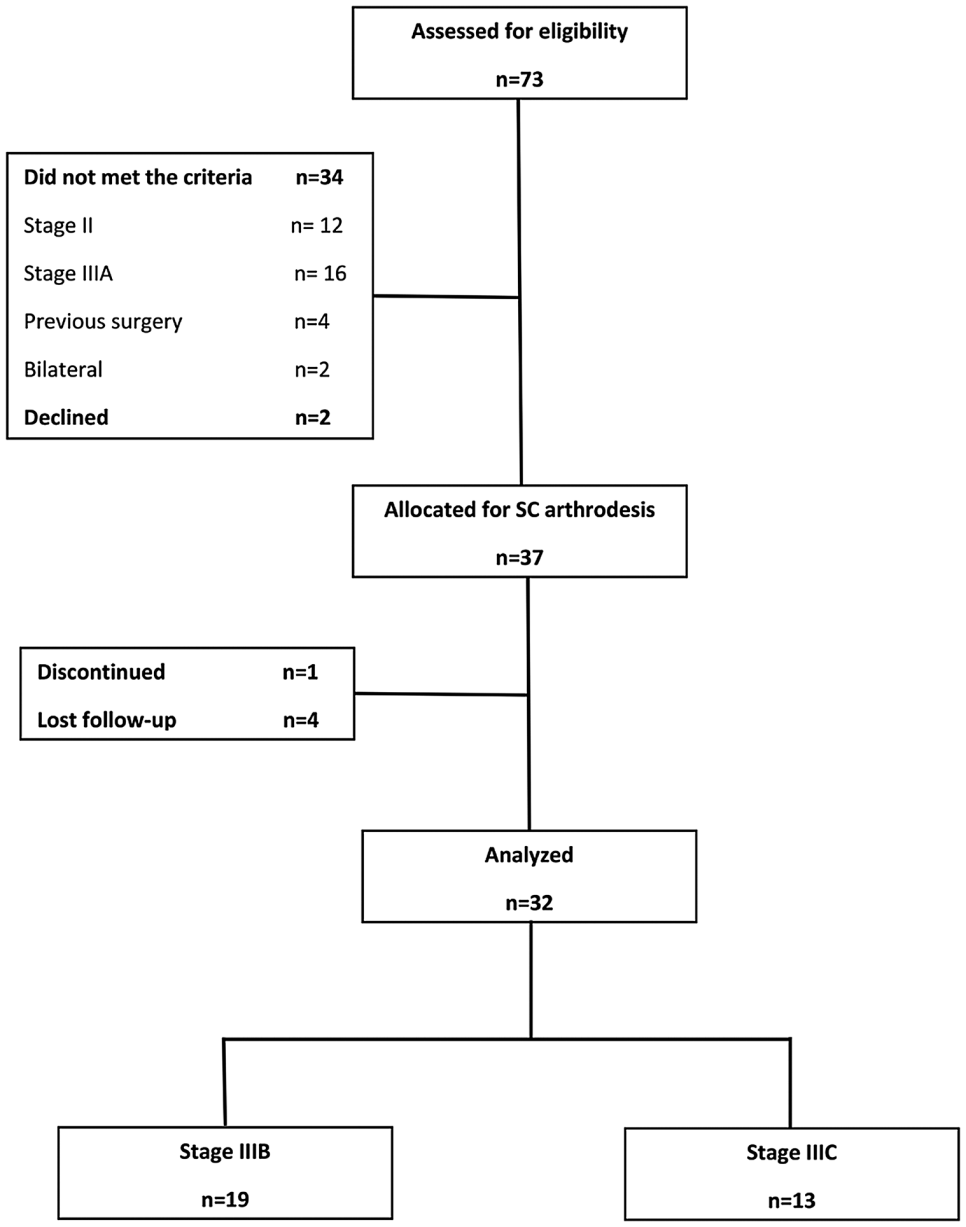
The study flowchart displays the count of included, excluded, and ultimately analyzed patients

The inclusion criteria were individuals over 18 years old diagnosed with stage IIIB or IIIC Kienböck’s disease, and having neutral ulnar variance. We excluded patients with bilateral disease, previous wrist surgery, or those with fewer than 12 months of follow-up. Diagnoses were confirmed through standard posteroanterior and lateral wrist radiographs, along with MRI imaging and CT scans. Out of the initial 73 patients, 36 were excluded for not meeting the criteria or declining to participate. The remaining 37 patients provided informed consent and underwent treatment discussions. Following surgery, one patient discontinued treatment and four were lost to follow-up. The final analysis comprised 32 patients with stage IIIB (*n* = 19) and IIIC (*n* = 13) Kienböck’s disease as depicted in the study flowchart (Fig. 1). Table 1 details the patient demographics.

**Table 1:** Patients’ demographics

Item	Stage of the disease		Total
	IIIB *n* (19)	IIIC *n* (13)	*n* (32)
Age, y	32.9 (19 to 48)	33.2 (19 to 48)	33 (19 to 48)
Gender (Male/Female), n	11/8	8/5	19/13
Occupation (manual/office workers/student/wives), n	11/4/1/3	7/3/1/2	18/7/2/5
Affected side (Right/Left), n	11/8	9/4	20/12
Dominant/nondominant, n	12/7	7/6	19/13
Smoking (yes/no), n	6/13	3/10	9/23

Data are given as mean (range) with 95% confidence interval

Two independent orthopaedic surgeons, each possessing level 3 experience [10], performed the clinical evaluations both before and after the surgery. They measured pain levels using a 10-point visual analogue scale (VAS) score [11], assessed total range of motion (ROM) using a two-hand goniometer, expressed as a percentage of the healthy side [12], and evaluated the single maximal effort of grip strength with a Jamar dynamometer, also expressed as a percentage of the healthy side, adjusting all measures for limb dominance [13]. Functional outcomes evaluation included the modified Mayo Wrist Score (MMWS) [14] and the Disabilities of the Arm, Shoulder, and Hand (Quick DASH) score [15]. Additionally, before and after surgery, two independent orthopaedic surgeons with level 3 experience carried out further radiographic evaluations. These assessments focused on the ulnar variance as measured by perpendiculars, radio-scaphoid (RS) angle, lunate height index (LHI) ratio, and carpal height index (CHI) ratio as measured by Youm [16, 17]. These evaluations also aimed to assess the union of SC arthrodesis and the presence of joint arthritis.

### Surgical technique

The procedure was carried out by either a senior author with level 4 experience [10] or a surgeon operating under his direct supervision. The patient underwent surgery while lying supine on the operating table, under either general or regional anaesthesia with a pneumatic tourniquet applied. An S-shaped dorsal wrist incision was made just ulnar to Lister’s tubercle, extending from the base of the second metacarpal to approximately 2 cm proximal to the tubercle (Fig, 2a), with meticulous avoidance of the radial sensory nerve branches. The extensor retinaculum was incised through the 3–4 extensor compartment, and the tendons were retracted [5] (Fig, 2b). The posterior interosseous nerve was identified and denervated.

**Fig. 2:**
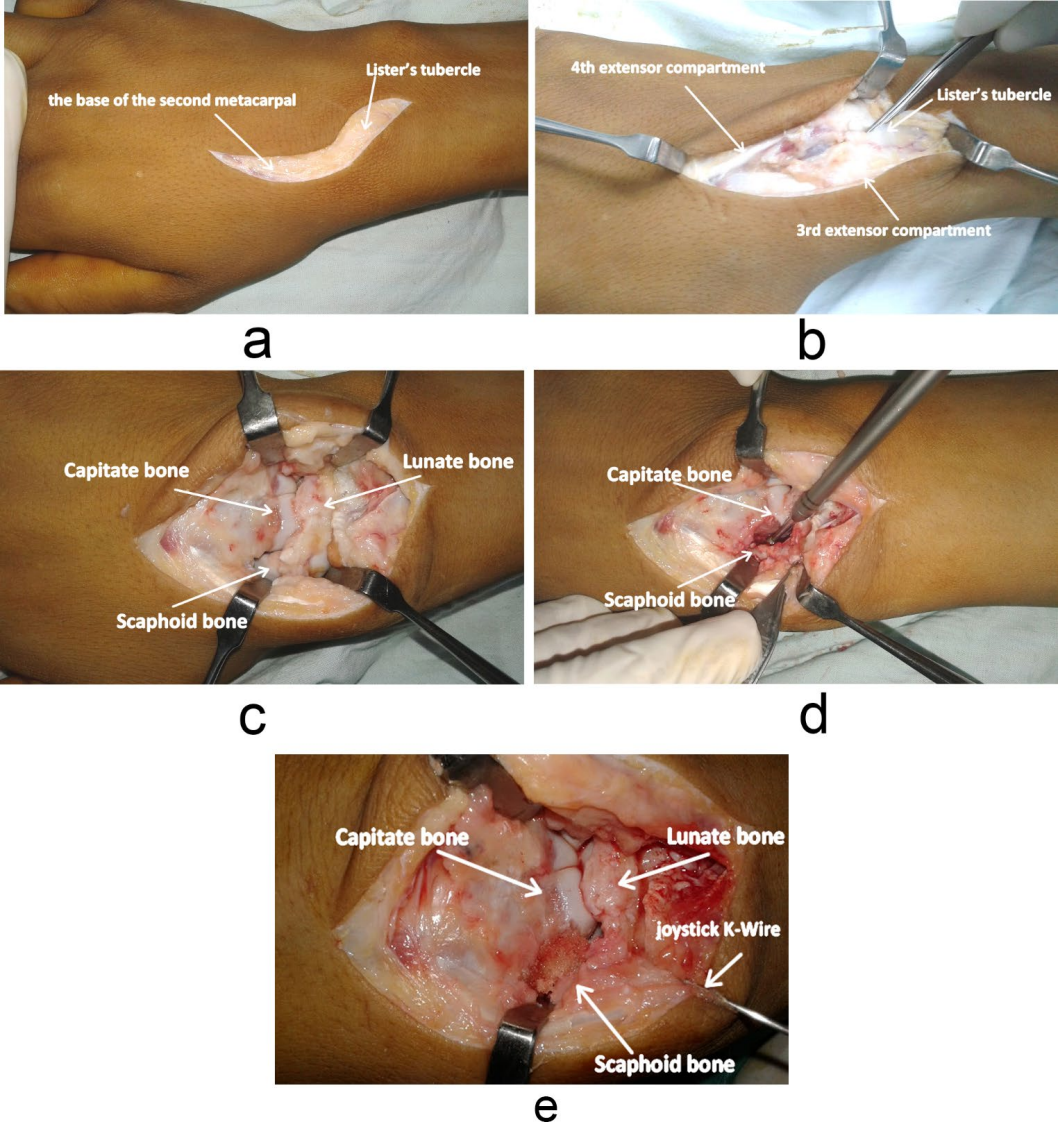
(**a**) An S-shaped dorsal wrist incision is made just ulnar to Lister’s tubercle, extending from the base of the second metacarpal to approximately 2 cm proximal to the tubercle. (**b**) The extensor retinaculum incised through the 3–4 extensor compartment. (**c**) The wrist capsule incised longitudinally, and the joint was explored. (**d**) Removal of SC articular cartilage while preserving the volar rim to maintain the space between the scaphoid and capitate bones. (**e**) Insertion of cancellous bone graft from the distal end of the radius into the SC joint

**Fig. 3:**
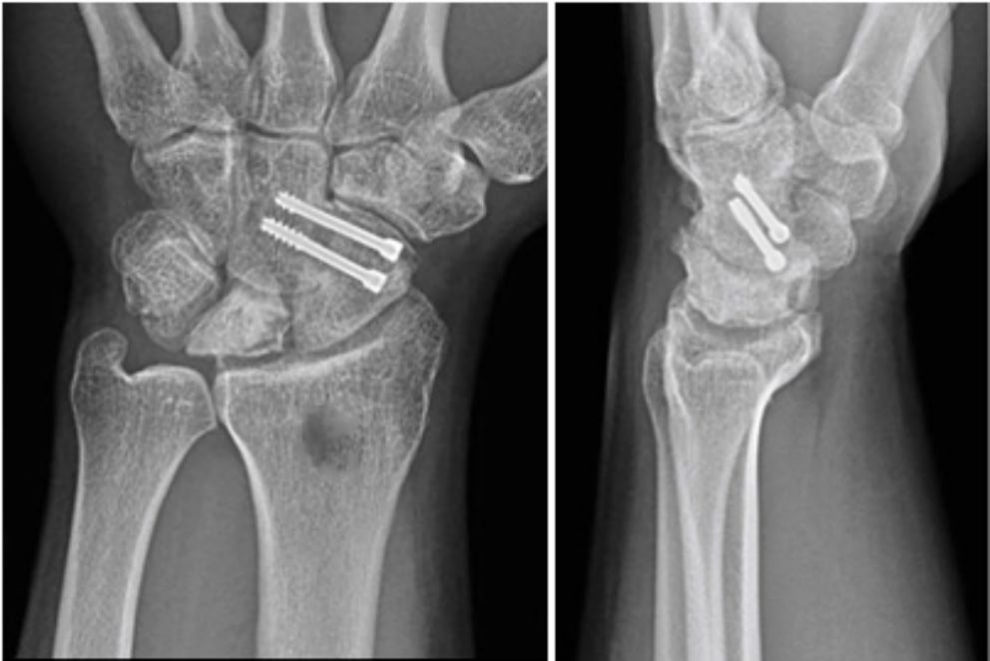
Radiographic posteroanterior and lateral views demonstrating union of the SC arthrodesis stabilized by two 3-mm HCS (Zimmer)

Using fluoroscopy, the SC joint was located and its wrist capsule was incised longitudinally and the joint explored ((Fig. 2c). The SC articular cartilage was removed while preserving the volar rim to maintain space between the scaphoid and capitate bones (Fig. 2d). K-wires were used as joysticks for correcting carpal misalignment and allowing the scaphoid to be released and derotated [8]. The repositioned scaphoid bone was temporarily stabilised with K-wires across the SC joint. A cancellous bone graft was taken from the distal end of the radius and inserted into the SC joint (Fig. 2e). The joint was secured under the guidance of image intensification using two 3-mm Herbert Compression Screws (HCS) (Zimmer), without compressing the SC joint (Fig. 3).

After deflating the tourniquet and ensuring haemostasis, the wound was irrigated and closed layer by layer. A sterile dressing was applied, and the limb was immobilised in a short-arm thumb plaster splint, set in slightly extended wrist and neutral deviation.

### Postoperative follow-up

The patients were provided with medications for pain control and advised to elevate their arms during the day to minimise swelling. They were encouraged to perform finger movements as well as elbow and shoulder exercises, which could be initiated immediately after surgery and repeated multiple times throughout the day. At the end of the second week, skin sutures were removed, and the plaster orthosis was replaced with a short-arm waterproof fibreglass cast with a thumb spica for continued support. By week eight, the cast was removed, and a removable orthosis was provided until radiographic confirmation of bone healing was obtained. Hand physical therapy sessions with a dedicated hand physiotherapist commenced, starting with gentle exercises and gradually progressing to more intensive wrist exercises. Once the union of the scaphoid bone was confirmed radiographically, patients were permitted to gradually resume heavy activities.

Two radiographic views of the wrist, including standard posteroanterior and lateral views, were taken biweekly until confirming union for SC arthrodesis. Union was assessed by the absence of a gap at the arthrodesis site or graft interface, no lucency around, shifting of the screws, or displacement of the graft visible on the radiographs. If union was suspected, wrist CT scans were used for confirmation. Additional CT scans were performed every three weeks if union was uncertain. Union confirmation was established when over 50% of trabecular bridging at the site or graft interface was visible on CT scans. Nonunion was identified by adverse features on radiographs or less than 50% trabecular bridging on CT at 24 weeks post-surgery.

Return-to-work decisions were tailored to the nature of the patient’s job. Patients with office jobs gradually resumed work while still in the cast, whereas manual labour was permitted post-union within pain tolerance limits. Full return to work and recreational activities were allowed post-union and alleviation of pain. Detailed records regarding the timing of full return to work and any complications were maintained throughout the treatment process.

### Statistical analysis

A sample size estimation was performed for the VAS and QuickDASH scores, identified as the primary outcome variables for the study. To achieve 95% power to detect a 10% difference at a significance level of *P* < 0.05, it was determined that 29 patients would be required for the VAS score and 23 patients for the QuickDASH score.

The outcome measures of all patients before surgery and at the final follow-up were compared using the paired sample t-test for continuous parametric variables and the Wilcoxon signed-rank test for continuous nonparametric variables. Similarly, the outcome measures of patients with stage IIIB and those with IIIC of the disease were compared before and after surgery using an independent t-test for continuous parametric variables and a Mann-Whitney U test for continuous nonparametric variables.

Categorical variables such as the rate of union, nonunion of SC arthrodesis, and complications were analysed using the chi-square or Fisher exact test. The values are presented as mean (range), with a 95% confidence interval. A p-value < 0.05 is considered statistically significant.

## Results

Before surgery, differences in RS angle, LHI, and CHI were observed between patients with stage IIIB and those with stage IIIC. However, no variations were found in pain levels (VAS score), ROM, grip strength, ulnar variance, MMWS, and QuickDASH scores between the two groups (Table 2).

**Table 2:** Preoperative clinical and radiographic parameters of patients with stage IIIB and IIIC Kienböck’s disease

Parameter	Stage IIIB	Stage IIIC	*P* value
VAS score (cm)	7.8 (6.5 to 8)	8.1 (7 to 9)	0.221
Total ROM (% of healthy side)	46.3% (41–49%)	45.2% (34–47%)	0.744
Grip strength (% of healthy side)	39.4% (39–42%)	39.6% ( 36–41%)	0.845
Ulnar variance (mm)	-0.5 (-2 to + 1)	-0.6 (-2 to + 1)	0.813
RS angle (degrees)	56° (54° to 61°)	60° (56° to 68°)	0.023*
LHI	0.43 (0.45 to 0.49)	0.41 (0.40 to 0.43)	0.042*
CHI	0.40 (0.39 to 0.45)	0.38 (0.36 to 0.39)	0.026*
MMWS	45 (40 to 50)	45 (35 to 50)	0.922
QuickDASH scores	77 (70 to 75)	79 (70 to 85)	0.781

VAS = Visual Analogue Scale, ROM = Range Of Motion, RS = Radio-Scaphoid, LHI = Lunate Height Index, CHI = Carpal Height Index, MMWS = Modified Mayo Wrist Score, QuickDASH = Disabilities of the Arm, Shoulder, and Hand. Data are given as mean (range) with 95% confidence interval. *p value is statistically significant

For all patients, the mean operative time was 73 min (67 to 75), and the follow-up period was 45.6 months (33 to 56). The union rate at the arthrodesis site was 91% (29 out of 32 patients), whilst the mean time to achieve union was 14 weeks (12 to 22). Additionally, the mean time to full return to work was 24 weeks (23 to 32). No differences were noted between patients with stage IIIB and those with stage IIIC regarding these parameters (Table 3).

**Table 3:** Follow up data and complications of patients with stage IIIB and stage IIIC Kienböck’s disease

Parameter	Stage of the disease		*p*-value
	IIIB *n* = 19	IIIC *n* = 13	
Time of surgery (minutes)	72 (67 to 75)	74 (70 to 75)	0.934
Follow-up period (months)	44.6 (33 to 56)	45.7 (33 to 56)	0.696
Time to union (weeks)	13.8 (12 to 22)	14.2 (12 to 22)	0.873
Union rate n (%)	17 (89.5%)	12 (92%)	0.561
Time to return to work (weeks)	24 (23 to 31)	25 (23 to 32)	0.612
Complications Infection (n) Hypertrophied sensitive scar (n) reflex sympathetic dystrophy (n) Nonunion (n) Scaphoid Impingement (n) degenerative changes (n)	2 1 0 2 0 5	0 1 1 1 1 3	0.361

Data are given as mean (range) with 95% confidence interval

There were significant differences from preoperative to postoperative in clinical outcomes (VAS score, grip strength) and radiographic measurements (RS angle) for all patients. Significant improvements were also observed in MMWS and QuickDASH scores. However, there were no differences in ROM, LHI, and CHI (Table 4). Furthermore, no postoperative differences in clinical or radiographic outcomes were found between patients with stage IIIB and those with stage IIIC, except for differences in LHI and CHI similar to the variations noted before surgery (Table 5).

**Table 4:** Pre- and postoperative clinical and radiographic parameters for all patients

Item	Preoperative	Postoperative	*p*-value
VAS score	8.2 (6.5 to 9)	1.3 (0 to 2.5)	0.001*
Total ROM (% of normal side)	46.8% (34–49%)	49% (44–51%)	0.161
Grip strength (% of normal side)	36% (33–39%)	79% (74–85%)	0.001*
RS angle (degrees)	59° (54° to 68°)	50° (46° to 60°)	0.001*
LHI	0.43 (0.45 to 0.49)	0.42 (0.45 to 0.49)	0.921
CHI	0.41 (0.36 to 0.46)	0.42 (0.40 to 0.48)	0.716
MMWS	45 (35 to 50)	75 (70 to 80)	0.001*
QuickDASH scores	78 (70 to 85)	21 (18 to 48)	0.001*

VAS = Visual Analogue Scale, ROM = Range Of Motion, RS = Radio-Scaphoid, LHI = Lunate Height Index, CHI = Carpal Height Index, MMWS = Modified Mayo Wrist Score, QuickDASH = Disabilities of the Arm, Shoulder, and Hand. Data are given as mean (range) with 95% confidence interval. *p value is statistically significant

**Table 5:** Postoperative clinical and radiographic parameters of patients with stage IIIB and IIIC Kienböck’s disease

Parameter	Stage IIIB	Stage IIIC	*P* value
VAS score (cm)	1.4 (0 to 2)	1.5 (1 to 2.5)	0.142
Total ROM (% of healthy side)	49.5% (34–50%)	48.3% (34–50%)	0.674
Grip strength (% of healthy side)	80% (74–91%)	79% (74–91%)	0.795
RS angle (degrees)	49° (46° to 54°)	54° (50° to 60°)	0.584
LHI	0.43 (0.45 to 0.49)	0.41 (0.40 to 0.43)	0.044*
CHI	0.43 (0.40 to 0.48)	0.42 (0.40 to 0.48)	0.039*
MMWS	75 (70 to 80)	75 (70 to 75)	0.931
QuickDASH scores	20 (18 to 48)	21 (18 to 48)	0.885

VAS = Visual Analogue Scale, ROM = Range Of Motion, RS = Radio-Scaphoid, LHI = Lunate Height Index, CHI = Carpal Height Index, MMWS = Modified Mayo Wrist Score, QuickDASH = Disabilities of the Arm, Shoulder, and Hand. Data are given as mean (range) with 95% confidence interval. *p value is statistically significant

Three patients had nonunion due to graft resorption and underwent revision surgery using an iliac crest cancellous bone graft and staples for fixation. Union at the arthrodesis site was achieved 11 weeks postoperatively. Two patients suffered superficial wound infections, managed with wound care and oral antibiotics. Additionally, two patients developed sensitive scar hypertrophy and were treated by a dermatologist. One patient received a diagnosis of reflex sympathetic dystrophy and underwent treatment involving physiotherapy, nonsteroidal anti-inflammatory drugs, and bisphosphonates [18]. One patient experienced radial side wrist pain due to impingement of the scaphoid and radial styloid, which was treated with radial styloidectomy. Five patients (23%) and three patients (13%) developed arthritic changes in the RS and scaphotrapeziotrapezoidal (STT) joints, respectively, but they were asymptomatic and required no treatment (Table 3).

## Discussion

**Fig. 4:**
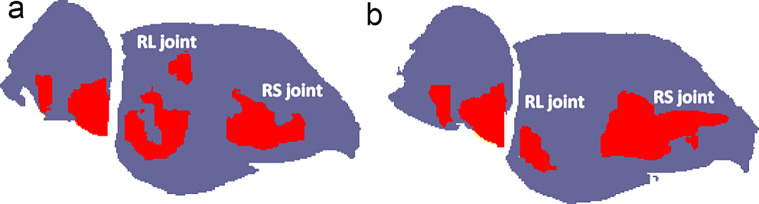
According to Iwasaki et al. [19] diagrammatic illustration showing load distribution (red areas) on the RS and RL joints; (**a**) Load distribution in the normal wrist. (**b**) Load distribution after SC arthrodesis

The rationale behind SC arthrodesis is that it reduces axial loading across the RL and lunocapitate (LC) joints while increasing the load through the RS joint. Additionally, it aims to correct or preserve scaphoid alignment and carpal height simultaneously. In an experimental biomechanical study by Iwasaki et al. [19] investigating limited intercarpal fusion for the treatment of Kienböck’s disease, they reported that SC and STT arthrodesis significantly decreased the joint force at the RL and LC joints compared with the intact wrist (Fig. 4). In contrast, these arthrodeses significantly increased the joint force at the RS joint in comparison with the intact wrist. Within the midcarpal joint, SC arthrodesis also increased the joint force at the STT and triquetral-hamate joints, whereas STT arthrodesis increased the joint force at the SC joint. Capitate-hamate (CH) arthrodesis resulted in no significant changes in the joint forces throughout the entire wrist joint. However, Gunal et al. [20] stated that no differences were found between CH arthrodesis and SC and STT arthrodesis above a load of 210 Newtons.

Several studies [1, 6–9] have presented evidence that SC arthrodesis significantly enhances grip strength. Notably, Pisano et al. [21] observed that the mean grip strength increased from 36% of the contralateral wrist’s strength preoperatively to 74% postoperatively. Similarly, Sennwald and Ufenast [22] reported that grip strength in the treated wrist improved from 37% preoperatively to 72% postoperatively relative to the healthy wrist. In contrast, the results of our current study showed an improvement in mean grip strength from 36% preoperatively to 79% postoperatively when compared to the healthy side. We believe that our structured rehabilitation program, guided by a specialised hand physiotherapist, was crucial in achieving superior grip strength outcomes.

A predictable loss of ROM is a known outcome following SC arthrodesis, though it is generally less severe than after STT arthrodesis [23]. Pisano et al. [21] observed significant reductions in the mean total ROM after SC arthrodesis, about 54% relative to the healthy side. Goyal et al. [6] treated 11 patients with advanced Kienböck’s disease and reported an overall decrease in the mean total ROM of 53%, compared to the contralateral wrist. Our findings mirror these, showing similar decreases in the total ROM by about 51% of the unaffected side. In contrast, Voche et al. [24] in their study on STT arthrodesis among 16 patients with Kienböck’s disease, noted reductions of 56% in the ROM. Additional studies [25, 26] on cadaveric specimens also indicated a greater loss of motion in simulated STT compared to SC arthrodesis. Despite the limitations in the ROM observed after SC arthrodesis, our study found that the restricted movement typically does not hinder the ability to perform daily activities. Significant improvements were noted in the mean values of the VAS score, which decreased from 8.2 preoperatively to 1.3 postoperatively (*p* = 0.001). Similarly, the MMWS experienced an increase from 45 preoperatively to 75 postoperatively (*p* = 0.001), and the QuickDASH score improved markedly from 78 preoperatively to 21 postoperatively (*p* = 0.001). Following the surgical intervention, all patients were able to return to their usual activities and employment with an average recovery period of 24 weeks. This demonstrates the procedure’s efficacy in maintaining functional outcomes despite the biomechanical changes induced by the surgery. Moy and Pemier [26] also reported that restriction of ROM following SC arthrodesis does not affect the ability to perform daily living activities.

In a study by Nakamura et al. [23], 20 patients with stages IIIA (4 patients), IIIB (10 patients), and IV (6 patients) of Kienböck’s disease were treated with either proximal row carpectomy (7 patients) or limited intercarpal arthrodesis (13 patients) including STT, SC, or RL arthrodesis. The study found that arthritic changes developed in adjacent joints in 39% of the patients initially treated with limited intercarpal arthrodesis, compared to 86% of those who underwent proximal row carpectomy. Our study indicated that approximately 36% of patients who underwent SC arthrodesis experienced arthritic changes in the RS or STT joints, with these arthritic changes predominantly occurring in older patients or those engaged in heavy manual labour. However, the changes were asymptomatic suggesting that while the surgery addresses primary issues, it requires careful monitoring of adjacent joints in certain patient groups. Theoretically, SC arthrodesis establishes a load-bearing column allowing forces to be transmitted from the hand to the distal radius while bypassing the lunate. However, locking the scaphoid to the distal carpal row can significantly reduce motion at the lunatocapitate articulation. This restriction may result in increased shear stress at the RL, RS, and STT joints, potentially accelerating the development of degenerative arthritic changes [2, 3].

The reported incidence of successful union at the site of SC arthrodesis ranges from 80 to 100%. Rhee et al. [1] and Pisano et al. [21] reported a union rate of 100% (27 patients) and 88% (15 out of 17 patients), at a mean of 19 and 22 weeks respectively. Their cases involved autogenous bone grafting (from the distal radius or iliac crest) and fixation with multiple K-wires, HCS, or staples. Similarly, Szalay et al. [27] observed an 80% union rate (24 out of 30 patients) with K-wire or HCS fixation, where successful revision arthrodesis was performed in five out of the six patients with nonunion. Our study’s results were in line with the existing literature, demonstrating a union rate of 92% (21 out of 23 patients) at a mean of 14 weeks (12 to 22). Upon identifying heavy smoking as a potential risk factor for nonunion in all patients who experienced this outcome, we implemented a precautionary measure mandating that they cease smoking before undergoing the revision surgery.

Watson et al. [28] suggested that the typical patterns of carpal collapse and scaphoid rotatory deformity seen in Kienböck’s disease are the primary causes of pain, limited motion, and synovitis in patients with this condition. In our study, we observed differences in radiographic measurements between patients with stage IIIB and those with stage IIIC, specifically in the RS angle, LHI, and CHI, preoperatively (Table 2) and in LHI and CHI postoperatively (Table 5). Despite these differences in radiographic parameters, no clinical significance was noted either pre- or postoperative between the two groups, indicating that these radiographic changes did not translate into significant differences in clinical outcomes. We propose that a critical factor contributing to the issues observed in Kienböck’s disease might be the rotatory instability of the scaphoid, which can lead to increased synovitis and subsequent pain, adversely affecting functionality. By stabilising the scaphoid through SC arthrodesis and performing a synovectomy, we have found that pain relief and functional improvements can be achieved regardless of whether there is a restoration of the CHI or LHI or not. This suggests that the key therapeutic benefit lies in stabilising the scaphoid and managing the inflammation rather than solely focusing on the anatomical alignment indexed by CHI and LHI. Minamikawa et al. [29] performed motion studies on cadaveric wrists, identifying an optimal RS angle range of 30° to 57° to enhance wrist kinematics after SC arthrodesis. In our own research, we noted a significant improvement in the mean RS angle, which decreased from 59° preoperatively to 50° postoperatively, with no changes in the LHI and CHI during the follow-up period. Similarly, Sennwald and Ufenast [22] reported an improvement in mean RS angles from 56° before surgery to 51° immediately after SC arthrodesis, sustained over a 51 months follow-up period. In contrast, Pisano et al. [21] found no significant loss of carpal height or scaphoid misalignment over a 23-month follow-up. However, Rhee et al. [1], who treated 27 patients with stage III (IIIA, 10; IIIB, 6) and stage IV (11 patients) Kienböck’s disease, reported a notable reduction in the mean CHI, indicative of progressive radiographic carpal collapse.

Several studies [1, 6–9] have explored adding lunate bone excision to limited intercarpal arthrodesis. Watson et al. [28] reported that 32% of patients might need lunate excision within two years following STT arthrodesis if the lunate is initially preserved. On the other hand, Lee et al. [30] pointed out a potential risk, noting that lunate excision could precipitate early degenerative changes in the RS joint, which presents a significant drawback of this procedure. In contrast, Luegmair and Saffar [31] observed in their small cohort that, although only four out of ten patients underwent lunate excision, there was a low incidence of degenerative changes over a nine-year follow-up. Similarly, Özdemir et al. [9] found that nine patients who underwent SC arthrodesis with lunate excision demonstrated satisfactory functional results over 17 months, without any reported degenerative changes. These findings highlight the variability in long-term outcomes of lunate excision in SC arthrodesis, underscoring the need for extended longitudinal studies.

Our study demonstrates that SC arthrodesis effectively relieves pain, enhances grip strength, and improves functionality in patients with advanced Kienböck’s disease, as evidenced by improvements in MMWS and QuickDASH scores. Additionally, the procedure aids in maintaining carpal height and restoring scaphoid alignment. However, it was observed that there are no differences in outcomes between patients classified as stage IIIB or IIIC Kienböck’s disease. This highlights the procedure’s consistent efficacy across these advanced stages of the disease.

## Data Availability

All raw data from this study are available.
